# Poor Kids? Economic Resources and Adverse Peer Relations in a Nationally Representative Sample of Swedish Adolescents

**DOI:** 10.1007/s10964-017-0747-8

**Published:** 2017-09-19

**Authors:** Simon Hjalmarsson

**Affiliations:** 1 The Swedish Institute for Social Research, Stockholm University, SE-106 91, Stockholm, Sweden; 2 Institute for Futures Studies, Holländargatan 13, SE-101 31, Stockholm, Sweden

**Keywords:** Peer rejection, Bullying victimization, Economic resources, Material deprivation, Poverty, Adolescence

## Abstract

There is limited knowledge on the impact of economic resources on adverse peer relations during adolescence. This study used a nationally representative sample (*n* = 4725, 51% girls) of Swedish eighth-grade students (approximately age fourteen) to examine associations between economic resources and adverse peer relations in the form of peer rejection and bullying victimization. Adolescents from households in the lowest within-school household income quintile were found to be rejected by school class peers to a greater extent than more advantaged students, but an association was not found between relative household income and bullying victimization. In contrast, adolescents unable to participate in activities with peers for economic reasons experienced more rejection and were at higher risk of victimization. The results underline the multidimensionality of adverse peer relations and advance our knowledge on how economic resources relate to peer relations in youth.

## Introduction

Adolescence is characterized by an increased importance of peers. At their best, peer relations serve as sources of support at a time of transitions and developmental changes. However, peer relations are far from equally rewarding to all. Some adolescents experience rejection by peers, and eight percent of fifteen-year-olds in the countries covered by the Health Behaviour in School-Aged Children (HBSC) survey report being victims of school bullying (Inchley and Currie 2016). Additionally, the risk of suffering adverse peer relations is not equal for all adolescents. The identification of individual and behavioral characteristics associated with higher risks of victimization has made significant contributions to understanding bullying and the development and targeting of intervention programs. However, less is known about the extent to which socioeconomic factors play a role.

If adolescents with worse economic conditions are at a higher risk of experiencing adverse peer relations, such as peer rejection or bullying victimization, this would represent yet another disadvantage facing this group of adolescents. Bullying victimization can have serious short- and long-term consequences (Arseneault et al. 2010; Wolke and Lereya 2015). Adverse peer relations could thus be one pathway through which economic disadvantage translates into socioeconomic inequalities in other dimensions. For instance, Due et al. (2009a) argued that social inequalities in early adulthood depression are partly explained by the differential exposure and impact of bullying victimization in adolescence.

This article examines the association between economic resources and adverse peer relations using data that are uniquely well-suited to this question, namely the Swedish part of the 2010 wave of the Children of Immigrants Longitudinal Study in Four European Countries (CILS4EU). This is a nationally representative sample of Swedish eighth-grade students (approximately age fourteen), containing a school-based survey directed to the adolescents, and matched information on household income and parental characteristics gathered from administrative and taxation register data. While previous research has predominantly examined the association between economic resources and adverse peer relations through measures of household economic resources measured in absolute terms, the current study applies an adolescent-centered perspective of economic resources: Economic resources are measured on (1) the household level, in terms of household income assessed relative to the household income of others in the same school, and (2) the individual level, in terms of adolescents’ self-reported economic and material deprivation. In addition, adverse peer relations are measured using two outcomes: Bullying victimization is measured through self-reports, while peer rejection is reported by school class peers and collected through sociometric (network) procedures. The use of two distinct forms of adverse peer relations, reported from different sources, allow for a more comprehensive understanding of the potential associations between economic resources and adverse peer relations.

### Peer Rejection and Bullying Victimization

There are several distinct forms of adverse peer relations, of which peer rejection and bullying victimization are two examples. Peer rejection describes a peer group’s attitude towards a member, that is, the degree of social avoidance, or reluctance to affiliate with, the individual student (cf. discussion in Juvonen and Gross 2005). It should be noted that, as a concept, peer rejection does not necessarily involve negative treatment by peers (cf. Schuster 2001). To be bullied, on the other hand, is to repeatedly and over time be the target of intentionally hurtful behaviors by peers when a power imbalance is also present, making it hard to defend oneself (Olweus 1993).

Although peer rejection does not always involve victimization (e.g., Knack et al. 2012), peer rejection and bullying victimization tend be fairly strongly correlated, especially when bullying victimization is measured through peer reports (Schuster 2001; Scholte et al. 2013). Peer rejection has also been found to predict victimization in longitudinal studies (e.g., Hodges and Perry 1999; Salmivalli and Isaacs 2005). Here, however, the purpose is not to examine links between peer rejection and bullying victimization but to examine whether economic resources are associated with either.

### Economic Resources and Adverse Peer Relations

Lacking the economic resources needed to participate in consumption and activities undertaken by peers could make it harder to create and maintain friendship relations. Indeed, poorer children and adolescents report having fewer friends (Olsson 2007; Sletten 2010), receive less friendship nominations from school peers (Bolger et al. 1995; Hjalmarsson and Mood 2015), and are at a higher risk of social isolation in the school class (Hjalmarsson and Mood 2015). Students with few friends, and especially those with no friends, in school are more likely to be victimized (Juvonen and Graham 2014), perhaps since bystanders are likely less prone to defend peers that they do not consider friends or with whom they do not want to be affiliated (cf. Hodges and Perry 1999).

Theories of relative poverty and deprivation suggest that economic inability to participate in common or expected activities or consumption generates feelings of shame and inadequacy, sometimes resulting in social withdrawal (e.g., Townsend 1979). Previous research has found both household economic resources and adolescents’ own experienced economic and material deprivation to be associated with internalizing symptoms and self-rated health (e.g., Plenty and Mood 2016). Internalizing problems (e.g., anxiety, insecurity in social interactions, and social withdrawal) are important risk factors for victimization (Juvonen and Graham 2014; Reijntjes et al. 2010).

Peers might also stigmatize adolescents from poorer backgrounds. Experimental research has found that person-group differences increase the risk of victimization (Wright et al. 1986; Boivin et al. 1995). When adolescents are asked to explain why bullying occurs, a common claim is that the victim deviates from group norms (Thornberg 2011; Thornberg and Knutsen 2011). A consistent finding from qualitative interviews with children in low-income families is the perceived importance of maintaining economic appearances in order to avoid being seen as different (Attree 2006; Ridge 2011). A lack of economic resources could itself form the basis for perceived deviation, but having less economic resources than others could also lead to deviation from group norms in other dimensions. For instance, poorer students may be construed as different based on “wrong” or out of fashion clothing (e.g., Frisén et al. 2008). Not being able to participate in normatively expected activities or consumption may lead to being seen by peers as “boring, cheap or weird” (Fernqvist 2013:79).

### Previous Research on Inequality in Rejection and Victimization

Scarce research attention has been given to whether economic resources are related to peer rejection. An early example is Patterson et al. (1990, 1991), who found that elementary school students from low-income families were twice as likely as other students to be rejected by school peers in a small American city. The association remained statistically significant after adjustment for ethnicity, family structure, and gender.

In comparison, more attention has been given to socioeconomic inequalities in bullying victimization. A recent meta-analysis (Tippett and Wolke 2014) found that students from lower socioeconomic backgrounds had increased odds of victimization but that the overall association, while statistically significant, was fairly weak. However, Tippett and Wolke (2014) also noted that a wide range of measures have been used to conceptualize socioeconomic background. Stronger associations have been found by studies using measures of household wealth. For instance, Due et al. (2009b) used HBSC-data on 11-, 13-, and 15-year-olds in 35 countries and found that lower scores on the Family Affluence Scale (FAS) were associated with a higher risk of victimization. Employing a similar measure, Chaux and Castellanos (2015) used data from a large-scale survey of Colombian students in the 5th and 9th grades and found that students from households with fewer assets than others in the same school class were at a higher risk of victimization. Weaker associations have been found by studies using measures of parental education or occupation (e.g., Nordhagen et al. 2005; Lemstra et al. 2012).

Only rarely have studies had access to information on household income. Olsson (2007) examined associations between household economic resources and a wide range of social outcomes—including victimization—using a nationally representative sample of Swedish 10–18-year-olds. Household income was associated with victimization, as was living in an economically vulnerable household (households with a low disposable income and no cash margin). However, after adjustment for parental and household characteristics (education, health, family structure, immigration background and type of region), associations were substantially reduced and no longer statistically significant.

These results indicate that victimization might not be affected by household economic resources per se but rather result from other factors associated with poorer economic conditions. The comparatively weak association between parental education and bullying victimization has already been mentioned, but other background factors, such as family structure, immigration background, parental health or unemployment, could also play a role. Students from single-parent households are more likely to report being victims of bullying in school (Fransson et al. 2017). Regarding immigration background, recent research in the Swedish context has reported mixed results. Hjern et al. (2013) found that first-generation immigrant students have a higher overall risk of victimization, while Plenty and Jonsson (2017) did not. However, both studies found the immigrant density of the school/school class to be of importance, indicating a more complex pattern of associations. Parental health problems may be associated with adolescents’ own health issues or with internalizing symptoms brought forth by the situation of having a sick parent. A similar argument can be made for parental unemployment.

### What Economic Resources Matter?

In the cases where previous literature has specifically studied the role of economic resources, the measures have almost exclusively been household-centered, referring to the household economic resources, and predominantly in absolute terms. Theoretically, this is unlikely to be the most appropriate choice when the outcome of interest is adverse peer relations in adolescence. First, insofar as parents’ resources matter, they are more likely to matter in relative than in absolute terms. During adolescence, school peers are likely to constitute the most salient reference group, and the economic conditions of peers are likely to determine what level of consumption and material resources are needed for participation and to avoid being perceived (or perceiving oneself) as deviating from group norms (cf. Bernburg et al. 2009). If adolescents from low-income households tend to attend schools in which their economic conditions are comparable to peers, associations between household income and adverse peer relations may not be evident if household income is measured in absolute terms. Adolescents with fewer economic resources relative to others in the same school could still be more likely to experience adverse peer relations.

Second, defining the adolescents’ economic situation in terms of the household economy renders the adolescents’ own economy invisible, and ignores the fact that the within-household distribution of resources may vary widely between families. Individual experiences of material deprivation can exist within more affluent households, and children in low-income households can sometimes be kept on material par with peers through parental self-sacrifice or support from outside the household (e.g., Kochuyt 2004). Research using parental reports (Main and Bradshaw 2014) and household expenditure surveys (Kornrich and Furstenberg 2013) indicate that parents in low-income households allocate larger shares of their income on the material conditions of their children. The link between household income poverty and child material deprivation has in fact been found to be fairly weak, at least in the Swedish context (Mood and Jonsson 2015). Household income could thus be too indirect to capture adolescents’ experienced material and economic conditions.

A more direct measure can be achieved using indicators of adolescent self-reported material and economic deprivation. Adolescents’ own economic and material conditions is what directly affects their opportunities for consumption and participation. For instance, lacking an own room could make it harder to bring home friends; an own cash margin allows for participation in spontaneous activities, or to make spending choices parents might not willingly provide funding for (e.g., following trends or fashion, buying snacks, lending money to a friend); and lacking access or control over material assets (e.g., a computer, TV, or phone with social functions), could bar from participation in activities, or complicate communication with peers. Adolescents’ own resources will also be observable to peers to a much larger extent than the economic resources of parents.

Measures of own resources can be both absolute and relative. Previous research suggests that children and adolescents who lack an own cash margin (absolute measure) or who are not able to afford what many others in the same age group can afford (relative measure) are at a higher risk of victimization, even after controlling for household economic resources and parental characteristics (Olsson 2007).

## The Current Study

The research presented above leads to the prediction that adolescents who are poorer than school peers are more likely to experience adverse peer relations. Thus, the first hypothesis states that adolescents from households with a lower disposable income relative to others in the same school are rejected by peers to a larger extent than their more affluent school class peers (Hypothesis 1). Relative household income is predicted to be associated with bullying victimization in the same way: Adolescents from households with a lower disposable income relative to others in the same school are more likely to be victims of bullying than their more affluent school class peers (Hypothesis 2).

Household income may be too indirect a measure to capture adolescents’ experienced material deprivation, because the link between parental and child economic conditions has been found to be fairly weak. Therefore, adolescents’ own economic and material conditions are expected to be associated with adverse peer relations independently of household income. The third hypothesis thus states that at a given household income, economically or materially deprived adolescents are rejected by peers to a larger extent than school class peers not experiencing deprivation (Hypothesis 3). The last hypothesis is that economic or material deprivation is associated with bullying victimization in a similar way: At a given household income, economically or materially deprived adolescents are more likely to be victims of bullying than school class peers not experiencing deprivation (Hypothesis 4).

## Methods

### Procedure and Participants

Survey data were gathered from the Swedish part of the first wave of the CILS4EU-project (Kalter et al. 2013).^1^As the main goal of the CILS4EU-project was to collect information on the social, cultural and structural integration of adolescents of immigrant descent, the survey was conducted in schools and used a two-step clustered sampling approach. Schools were randomly selected within four strata based on the proportion of students with immigration background, oversampling schools with a higher proportion. Within the selected schools two school classes were randomly chosen for participation in the survey (CILS4EU 2016). Survey weights account for the sampling probabilities and allow inferences about the 2010/2011 cohort of Swedish eighth-grade students (approximately age fourteen).

Statistics Sweden (the Swedish government statistics agency) collected the data in schools during the winter of 2010–2011. Respondents were asked to answer questionnaires and to complete tests on language proficiency and cognitive skills. In addition, sociometric (network) data were collected: students were asked to nominate other students in their class for questions of interpersonal relationships (e.g., “Who do you not want to sit next to?”). After ethical approval, Statistics Sweden linked the survey to information drawn from register data on household income and parental characteristics.

The original sample consisted of 5699 students in 251 classes and 129 schools. Students absent at the time of the survey (*n* = 674) were removed. Twenty-six students were omitted due to implausible responses on multiple items, as were the sociometric nominations they made. To reliably measure economic conditions in the school, schools where less than 30 students had valid information on income were excluded (affecting 14 schools, 18 school classes and 274 students), mostly affecting schools with fewer than 30 participating students. The analytical sample thus consisted of 4725 students in 233 classes and 115 schools.

The analytical sample was evenly distributed in regards to gender (51% female) and consisted of 69% students of majority background (Swedish born students with at least one Swedish born parent), 20% of students had a second generation immigration background (born in Sweden to foreign born parents), and 11% of students had a first generation immigration background (born outside Sweden to foreign born parents). Among students with a first or second generation immigration background 44% had a Middle Eastern or North African background, 25% an Eastern European background, 13% was of Sub-Saharan African descent, 8% had an Asian background, 5% had a Southern or Western European background, and 5% descended from other regions or could not be classified.

For the analysis of peer rejection a sub-sample was used. Since sociometric data are sensitive to nominator non-response 33 school classes in which less than 70% of students answered the question used to measure rejection were excluded; a response rate lower than approximately this level has been suggested as problematic (e.g., Cillessen and Marks 2011; but see also Marks et al. 2013). The sub-sample for peer rejection was thus reduced to 4106 students in 200 classes and 112 schools.

### Measures

#### Outcomes

##### Peer rejection

The measure of peer rejection was based on the number of received sociometric nominations on the question, “Who do you not want to sit next to?”. Students were allowed to make up to five nominations. Nominations where the nominated student was simultaneously reported as a friend were removed (185 students made 331 such overlapping nominations, representing approximately 3% of the total nominations given). To account for differences in class size and for class differences in nomination propensities, the number of received nominations were standardized by subtracting the school class mean of received nominations and dividing the remainder by the school class standard deviation (Newcomb and Bukowski 1983), thus expressing the difference from the school class mean in standard deviations. In order to facilitate the interpretation of the results, the standardized measure was then multiplied by the overall sample standard deviation. The resulting measure can be interpreted as the number of nominations received by the student relative to the school class mean. Continuous sociometric rejection measures have been found to have good test-retest reliability (Jiang and Cillessen 2005).

##### Bullying victimization

Bullying victimization was based on three questions in which the respondents were asked to think about the past month and report how often (every day, one or several times a week, less often, never) they had been afraid of, teased by, or bullied by other students. Adolescents experiencing any of these events at least once a week were considered victimized, as were students experiencing all of the above events on a monthly basis.

#### Predictors

##### Relative household income

Household income quintiles were created within schools and were based on taxation register data on the disposable income of the household (income from labor, capital, and social benefits available to the household after taxation) in 2010. In cases where custodial parents were registered as living in separate households, the mean of the two households was used. Adolescents in the middle within-school income quintile was used as the reference category.

##### Misses out on activities

This item was measured based on the question: “How often do you miss out on activities your friends do because you can’t afford it?” Students reporting that they often or always experience this problem were coded as 1, while all others were allocated to the reference category.

##### Lack of own cash margin

Adolescents able to access 300 SEK (approximately $30) by the next day were seen as having a *cash margin* and coded as the reference group, while adolescents who were not able to do so or who answered that they might be able to do so were seen as lacking a cash margin.

##### Lack of own room

Adolescents reporting to have their own room for the question: “Do you have a room just for yourself?” was coded as the reference category, while 1 refers to not having one’s own room.

##### Own material assets

Material assets were measured as the sum of material assets the adolescent reported to own (out of own computer, TV, smartphone, and having a gaming console in the household).

#### Control variables

##### Parental education

Parental education was coded as the highest achieved educational degree of the parent(s) in 2010. It was based on register data and distinguishes between parents with at most a junior high school degree (högstadieexamen), a senior high school degree (gymnasieexamen), and parents with a higher educational degree (eftergymnasial utbildning).

##### Family structure

Family structure was distinguished between adolescents whose parents reside in the same household (i.e., who have parents that are married or cohabiting) and all other family forms. The variable was based on register data from 2010.

##### Migration background

In regards to migration background, adolescents of majority background (i.e., having at least one Swedish-born parent) formed the reference category. Further distinctions were made between second-generation immigrants (born in Sweden to foreign-born parents), first-generation immigrants (born abroad to foreign-born parents) having migrated to Sweden more than 2 years prior to the interview, and first-generation immigrants having lived in Sweden for less than 2 years. Adolescents’ place of birth was assessed using register data, as was the time since migration. Parents’ country of birth was determined based on information provided by the students.

##### Parental receipt of disability pension

Parental receipt of disability pension was collected from register data and indicates that at least one parent received disability pension during the year 2010. Disability pension compensates for income reductions caused by a permanently reduced working ability due to sickness, injury or disability.

##### Parental unemployment

The adolescent was seen as having an unemployed parent if register data indicated that at least one parent had been unemployed for more than half of the year (during 2010).

##### Gender

Gender used boys as the reference category and was based on self-reported survey data.

##### Age

Age was measured in days but expressed in years and centered on the sample mean. The information came from the student questionnaires.

##### Cognitive test score

Cognitive test score was coded as the sum of correctly answered questions (0–30) on a cognitive test that was carried out as part of the survey. This sum was then centered on the school class mean. A non-centered version of the variable was included as an auxiliary variable in the multiple imputation models (see section on Item non-response below).

##### Language test score

The language test was exclusively used as an auxiliary variable in the multiple imputation models. The variable is based on a test of language proficiency (in Swedish), where the scores ranged from 0–30.

##### Externalizing behavior

Externalizing behavior (scale 0–3, Cronbach’s alpha .70) was measured as the mean score of eight items. The index was created based on four questions asking whether the respondent, during the last 3 months, had “Deliberately damaged things that were not yours”, “Stolen something from a shop/from someone else”, “Carried a knife or weapon”, or “Been very drunk”; and four questions where respondents reported how often (every day, once or several times a week, once or several times a month, less often, never) they “argue with a teacher”, “get a punishment in school”, “skip a class”, or “come late to school”.

##### Internalizing problems

Internalizing problems (scale 0–3, Cronbach’s alpha .78) was measured as the mean score on three items regarding how often the statements “I feel very worried”, “I feel anxious”, and “I feel depressed” was true about the student (never true, rarely true, sometimes true, often true).

### Item Non-response

Table 1 presents (unweighted) descriptive statistics for the analytical sample. While the use of administrative register data means that few observations lack information on household characteristics, approximately 16% (*n* = 738) of observations in the analytical sample lack a response on at least one of the questions concerning adolescents’ own economic resources (see proportion for each analysis variable in Table 1). For this reason, multiple imputation by chained equations (MICE) was used to impute missing values. The mi impute command in Stata 14.2 was used to generate 20 imputed datasets. Imputation models included all variables used in the respective analysis. Additionally, since non-response was judged to mainly be a problem of respondent fatigue (other questions late in the survey also suffered low response rates), non-centered test scores from the cognitive ability and language proficiency test were included as auxiliary variables in both imputation models. Imputation models were stratified by gender to allow the analyses to assess interactions with gender. Cases with missing information on bullying victimization (*n* = 123) were included in the imputation model for bullying victimization, but excluded from the analysis, consistent with recommendations for missing data on outcome variables by Von Hippel (2007). Similarly, observations with missing data on household income and parental education were used in imputation models but excluded from analyses since the imputation model was not developed to account for the missing data mechanism of the missing register data. The same approach was used for observations lacking information on the cognitive test score used in the analysis of peer rejection (see Analytical Approach below). Models using casewise deletion are available in the Appendix (Table 3). The results are substantively similar.

**Table 1:** Unweighted descriptive statistics of the analytical sample (*n* = 4725)

Outcomes	Freq. (%)		Freq. (%)	Mean (Std. D.)
Bullying victimization		Peer rejection (std. and scaled)^a^	4106 (86.9)	−0.11 (2.40)
Not victimized (ref.)	4165 (88.1)	Missing	619 (13.1)	
Victimized	437 (9.3)			
Missing	123 (2.6)	Peer rejection (raw)^a^	4106 (86.9)	1.77 (2.50)
		Missing	619 (13.1)	
Predictors				
Relative household income		Cash margin		
Quintile 1	966 (20.4)	Has cash margin (ref.)	2841 (60.1)	
Quintile 2	923 (19.5)	Lacks cash margin	1330 (28.2)	
Quintile 3 (ref.)	940 (19.9)	Missing	554 (11.7)	
Quintile 4	947 (20.0)			
Quintile 5	920 (19.5)	Own room		
Missing	29 (0.6)	Has own room (ref.)	3511 (74.3)	
		Lacks own room	572 (12.1)	
Misses out on activities		Missing	642 (13.6)	
Does not miss activities (ref.)	3878 (82.1)			
Misses activities	300 (6.3)	Own material assets (0–4)	4034 (85.4)	2.59 (1.05)
Missing	547 (11.6)	Missing	691 (14.6)	
Control variables				
Family structure		Parental unemployment		
Living with both parents (ref.)	2959 (62.6)	Not unemployed (ref.)	4351 (92.1)	
Not living with both parents	1766 (37.4)	Unemployed	374 (7.9)	
Missing	0 (0.0)	Missing	0 (0.0)	
Parental education		Gender		
Comprehensive or lower	381 (8.1)	Boy (ref.)	2321 (49.1)	
Upper secondary (ref.)	2069 (43.8)	Girl	2404 (50.9)	
University	2222 (47.0)	Missing	0 (0.0)	
Missing	53 (1.1)			
		Age at interview	4725 (100.0)	14.7 (0.38)
Migration background		Missing	0 (0.0)	
Majority (ref.)	3232 (68.4)			
Second generation	958 (20.3)	Age at interview (centered)	4725 (100.0)	0 (0.38)
First generation, ≥ 2 years	490 (10.4)	Missing	0 (0.0)	
First generation, <2 years	45 (1.0)			
Missing	0 (0.0)	Cognitive test	4466 (94.5)	17.72 (4.83)
		Missing	259 (5.5)	
Parental disability pension				
No disability pension (ref.)	4281 (90.6)	Cognitive test (centered)	4466 (94.5)	0.02 (4.41)
Disability pension	444 (9.4)	Missing	259 (5.5)	
Missing	0 (0.0)			
		Language test	4512 (95.5)	18.4 (5.15)
		Missing	213 (4.5)	

^a^ Descriptive statistics for peer rejection refers to the rejection sub-sample. Missing refers to respondents in excluded school classes

### Analytical Approach

Associations between economic resources and peer rejection were analyzed using ordinary least squares regressions. All models analyzing rejection included a control variable for cognitive ability (centered on the school class mean) due to the phrasing of the question used to construct the rejection measure (“Who do you not want to sit next to?”). The worry is that the measure may entail aspects of academic ability, making the student more or less attractive to sit next to (e.g., inability to help during class).

Associations between economic resources and victimization were analyzed using logistic regression. Estimates are presented as Average Marginal Effects (AME), which—as opposed to odds ratios—allow for comparisons across models (e.g., Mood 2010). AME can be interpreted as the average percentage point change in the dependent variable associated with a one-unit increase in the independent variable.

For both outcomes, three sequential models were used. The first model examines relative household income. The second model adjusts for parental characteristics. The third model adds measures of own experienced economic and material deprivation. All models also adjust for age (expressed in years from the sample mean) and gender. Analyses were made using Stata 14.2. Official survey weights were used to account for the over-sampling of schools with larger proportions of students with immigration backgrounds. Standard errors are clustered (and heteroscedasticity robust) on the school class level.

Although the employment of control variables here is fairly extensive, it is unlikely to successfully adjust for all parental characteristics that may lead to confounding. As a robustness test, models including self-reported internalizing and externalizing behavior were also performed. The argument is that, after adjustment for the parental characteristics included in the models, most of the remaining unobserved parental characteristic should affect the risk of rejection and victimization through characteristics and behaviors of the adolescent (because peers are not very likely to observe parental characteristics directly). Thus, if an association remains after adjusting for internalizing and externalizing behavior, this provides stronger support for the hypothesis that there is an effect of household economy. However, since internalizing and externalizing behaviors are also likely to (1) be a mediating path for effects of economic resources and (2) be outcomes of victimization and rejection (i.e., they are endogenous to the model), these analyses should be considered only as a robustness test.

## Results

### Descriptive Statistics

Among responding students in the analytical sample, the (weighted) proportion of students reporting to miss out on activities with peers for economic reasons was 7%. A substantially larger proportion (32%) lacked a cash margin, 8% reported to lack an own room, and the average number of own material assets was 2.6. The measures of own material deprivation were, at most, weakly correlated (r ranging between −.17 and .15). Turning to the (weighted) prevalence of adverse peer relations, 9.5% of students were classified as victimized and the average number of received rejection nominations (using the peer rejection sub-sample) was 1.8. The measures of adverse peer relations were weakly correlated (r = .14, *p* < .01). Bivariate correlations between all outcome and predictor variables are available in the Appendix (Table 4) and unweighted sample characteristics are shown in Table 1 (above).

### Results for Peer Rejection

We begin by analyzing associations between economic resources and peer rejection, where the outcome is the received number of rejection nominations relative to the average number of nominations received by other students in the same class. Table 2 (Model 1) shows the association between relative household income and rejection, controlling for age, gender, and cognitive ability. Students in the lowest within-school household income quintile received 0.7 more rejection nominations (0.742, CI 0.451–1.032) than students in the middle-within-school household income quintile. Additionally, students in the second-lowest within-school household income quintile received more peer rejection nominations (0.370, CI 0.065–0.674) than students in the middle-within-school household income quintile. Adolescents from households in the highest and second-highest within-school household income quintiles received slightly fewer rejection nominations than the reference category, but in contrast to the lower within-school household income quintiles, the differences were not statistically significant.

**Table 2:** Regression of received rejection nominations (Models 1–3) and the probability of bullying victimization (Models 4–6) on relative household income, own economic and material deprivation, and control variables. Models 1–3 give OLS regression coefficients, whereas Models 4–6 give average marginal effects from logistic regressions

	Peer rejection (*n* = 3895)			Bullying victimization (*n* = 4548)		
	Model 1	Model 2	Model 3	Model 4	Model 5	Model 6
Household income (ref., Quintile 3)						
Quintile 1	0.742**	0.551**	0.551**	0.009	−0.011	−0.011
	(0.147)	(0.163)	(0.163)	(0.017)	(0.017)	(0.017)
Quintile 2	0.370*	0.283	0.271	0.015	0.004	0.000
	(0.154)	(0.158)	(0.157)	(0.018)	(0.019)	(0.018)
Quintile 4	−0.044	0.006	0.010	0.009	0.014	0.016
	(0.134)	(0.135)	(0.133)	(0.017)	(0.018)	(0.018)
Quintile 5	−0.128	−0.034	−0.026	−0.004	−0.001	0.002
	(0.149)	(0.151)	(0.153)	(0.016)	(0.016)	(0.016)
Not living with both parents		0.054	0.033		0.033**	0.028*
		(0.123)	(0.124)		(0.012)	(0.012)
Parental education (ref., Upper secondary)						
Comprehensive school or lower		0.225	0.191		−0.005	−0.007
		(0.249)	(0.249)		(0.026)	(0.024)
University		−0.224**	−0.205*		0.007	0.009
		(0.080)	(0.079)		(0.012)	(0.012)
Migration background (ref., Majority)						
Second generation		0.298*	0.258		−0.044**	−0.042**
		(0.126)	(0.139)		(0.012)	(0.013)
First generation, ≥2 years		0.357	0.245		0.015	0.004
		(0.189)	(0.212)		(0.025)	(0.022)
First generation, <2 years		2.337**	2.304**		0.126	0.122
		(0.454)	(0.463)		(0.132)	(0.123)
Parent has disability pension		0.495*	0.457*		0.041	0.031
		(0.194)	(0.194)		(0.027)	(0.024)
Parent is unemployed		0.346	0.328		0.002	0.001
		(0.183)	(0.185)		(0.021)	(0.021)
Misses activities due to lack of money			0.673**			0.109**
			(0.253)			(0.027)
Adolescent lacks cash margin			0.092			0.040**
			(0.097)			(0.011)
Adolescent lacks own room			0.267			0.007
			(0.216)			(0.024)
Own material resources (0–4)			0.017			−0.006
			(0.047)			(0.006)
Cognitive test score (centered)	−0.068**	−0.060**	−0.058**			
	(0.011)	(0.011)	(0.011)			
Girl	−0.312**	−0.312**	−0.306**	−0.009	−0.009	−0.013
	(0.117)	(0.116)	(0.117)	(0.011)	(0.011)	(0.012)
Age at interview (centered)	−0.173	−0.224	−0.230	0.003	0.002	0.002
	(0.125)	(0.125)	(0.124)	(0.015)	(0.015)	(0.014)
Constant	−0.175	−0.192	−0.322			
	(0.120)	(0.127)	(0.178)			

Robust standard errors in parentheses

*p < 0.05, **p < 0.01

Adjusting for parental characteristics (Table 2, Model 2) reduced the strength of the association between relative household income and peer rejection. Being from a household in the lowest within-school household income quintile was associated with receiving 0.6 more rejection nominations (0.551, CI 0.230–0.873) than being from households in the middle quintile. Students in the second-lowest within-school household income quintile received 0.3 more peer rejection nominations (0.283, CI -0.028–0.595), no longer being a statistically significant difference from the reference category. Confounding in the form of parental characteristics thus explains at least part of the association between relative household income and peer rejection. It is noteworthy that adolescents who migrated to Sweden less than 2 years prior to the interview received large numbers of rejection nominations. The group, however, is small (*n* = 45), and excluding them from the analyses does not change results (not shown).

The inclusion of variables measuring adolescent experienced economic and material deprivation (Table 2, Model 3), only slightly changed the relative household income quintile coefficients. Adolescents missing out on activities with friends because they could not afford to participate received, on average, 0.7 (0.673, CI 0.174–1.172) more rejection nominations than students who did not experience this problem. Lacking an own cash margin, lacking an own room, and the number of own material assets were not significantly associated with the number of rejection nominations received. Exploratory analyses in which gender was interacted with economic resources showed that economic and material resources appear to be somewhat more important for girls than for boys (not shown).

In summary, adolescents in the lowest within-school household income quintile were found to be rejected by peers to a greater extent than more advantaged students, even after adjustment for household and parental characteristics (Hypothesis 1). This was also the case for adolescents who missed out on activities with peers due to a lack of economic resources. Since an association was not found for all measures of own experienced economic and material deprivation, the results provide only partial support for Hypothesis 3. Nevertheless, relative household income and at least some aspects of own experienced deprivation are associated with peer rejection.

### Results for Bullying Victimization

We now turn to analyses of bullying victimization. No association between relative household income and victimization was found (Table 2, Model 4), and this remained the case after adjusting for parental characteristics (Table 2, Model 5). Adding measures of adolescent experienced economic and material deprivation (Table 2, Model 6) only marginally affected the coefficients for relative household income. However, adolescents reporting missing out on activities for economic reasons were 11 percentage points (0.109, CI 0.057–0.161) more likely on average to be victimized in school than students who did not experience this problem. Lacking a cash margin was associated with a 4-percentage-point greater probability of victimization (0.040, CI 0.018–0.062). Given that the average (weighted) risk in the sample was 9.5%, this represents a large increase in the probability of bullying victimization. In contrast, no statistically significant association was observed for lacking an own room or for the number of own material assets. No association was found to be statistically significantly moderated by gender (not shown).

Thus, no evidence of a relationship between relative household income and victimization was found (Hypothesis 2), while measures of adolescents’ experienced economic deprivation—in the form of lacking an own cash margin and reporting missing out on activities with peers for economic reasons—were associated with a substantially higher risk of victimization. Since no association was found with the number of own material assets, or with having one’s own room, the hypothesis that own experienced economic and material deprivation plays a role again finds only partial support (Hypothesis 4).

### Robustness Tests

The results are robust to alternative specifications of relative household income (not shown). The results were substantively similar when relative household income was measured continuously (centered on the school mean), and when non-linearity were examined using within-school household income deciles instead of within-school household income quintiles. Neither were conclusions altered when models using equivalized household income (dividing the income by the square root of the number of parents and children in the household) were performed. As information on the number of residents in the household was only available through survey data, the use of equivalized household income introduces additional non-response. Non-equivalized versions of relative household income were thus preferred.

Alternative specifications of the outcome variables have also been tried (not shown). For the measure of peer rejection, versions where the received number of nominations was top-coded to two or three standard deviations from the within-school-class mean before standardization led to similar conclusions. Non-standardized versions also show substantively similar results. For the measure of bullying victimization, not including students experiencing all of the events on a monthly basis decreases the number of victimized students, but does not alter conclusions.

As an additional robustness test, analyses adjusting for adolescent reported problems and behaviors were performed (see Appendix, Table 5), which, in general, somewhat reduced the strength of associations, especially for measures of adolescent experienced economic and material deprivation. However, all previously observed associations remained statistically significant, meaning that, even when we compare adolescents with equal behavior (in the measured respects), those with worse economic conditions still have worse outcomes. This result reduces the number of plausible confounding factors, as these factors would have to have an impact through other pathways than the externalizing or internalizing behaviors of the adolescent.

## Discussion

Experiences of adverse peer relations in adolescence, such as peer rejection and bullying victimization, can have serious short- and long-term consequences (Arseneault et al. 2010; Wolke and Lereya 2015). While risk factors connected to personal and behavioral characteristics have received substantial research attention, less is known about the extent to which socioeconomic factors play a role and, more specifically, whether economic resources are associated with the risk of experiencing adverse peer relations. Previous research examining this association have predominantly used household economic resources measured in absolute terms. In contrast, this article takes an adolescent-centered perspective, arguing that the economic resources likely to have repercussions for adverse peer relations are those that influence the ability to participate in consumption and activities on par with peers. Thus, household income was assessed relative to others in the same school, and self-reported indicators of economic and material deprivation were used to capture adolescents’ own experienced economic conditions.

The results showed relative household income to be associated with peer rejection. Adolescents from households in the lowest within-school household income quintile were—also after adjustment for potentially confounding factors—rejected by school class peers to a greater extent than more advantaged students. Previous findings have shown an association also between relative household income and the number of friends (Hjalmarsson and Mood 2015), which suggests that relative household income may have an effect on both positive and negative dimensions of peer relations during adolescence—perhaps because growing up in a household with less economic resources than others could make it difficult to reach normative expectations in one’s peer group.

On the other hand, relative household income does not appear to matter for the risk of bullying victimization. This finding is largely in line with previous studies using absolute measures of household economic resources (Olsson 2007), as well as with studies of various measures of socioeconomic background, which, in general, have found only weak associations with bullying victimization (Tippett and Wolke 2014). Overall, it seems unlikely for measures of household economic resources—whether measured absolutely or relative to others in the same school—to have more than marginal effects on the risk of bullying victimization.

That relative household income was found to be associated with peer rejection, but not with bullying victimization, suggests that a low economic standing may yield lower peer status, but that this lower status is not necessarily translated into the more severe outcome of being bullied. It has been suggested that peer rejection implies a distancing that could be interpreted by potential perpetrators as a group sanctioning to target the rejected student, and that it might reduce the likelihood of bystanders to intervene in bullying situations (Hodges and Perry 1999). However, although peer rejection is likely to form part of the process leading up to victimization, economic resources is only one factor out of a larger set of risk factors for peer rejection, and the risk for bullying victimization is, in turn, also affected by many other circumstances (e.g., Kärnä et al. 2010). More research is needed on how risk factors relate to different forms of adverse peer relations, and on how risk- and protective factors combine to make students more or less vulnerable to becoming targets of bullying behavior (e.g., Knack et al. 2012), but household income is unlikely to be of any major importance for the risk of experiencing bullying victimization.

It should however be added that this study examined associations between relative household income and adverse peer relations on the individual level and within schools. There is an additional need for research focusing on whether economic characteristics on the school level, such as the economic conditions of the student body or the within-school economic inequality (e.g., Elgar et al. 2009), are associated with the prevalence of bullying victimization on the school level.

When it comes to own experienced economic and material deprivation, adolescents missing out on activities with peers for economic reasons were rejected to a larger extent and had a substantially higher risk of victimization. Adolescents lacking an own cash margin were also in higher risk of victimization. These findings are in line with previous research on links between children’s and adolescents’ own experienced material deprivation and negative outcomes ranging from psychosomatic complaints to social isolation and bullying victimization (Plenty and Mood 2016; Hjalmarsson and Mood 2015; Olsson 2007).

Not all aspects of own experienced economic and material deprivation were associated with adverse peer relations, which could indicate that the impact of economic resources is more detrimental when it affects specific aspects of social relations, such as the ability to participate in activities, rather than when affecting ownership of material assets. However, it should be acknowledged that the measure of own material assets was fairly crude. In addition to mere ownership, the brand, version, or specific functions could have additional impact. It is also possible that, for instance, lacking one’s own room could matter more in areas where access to an own room is the norm, than in areas where it is more common. More research from a child-centered perspective of economic resources thus appears to be needed. The links between household income and child experienced economic and material deprivation have been found to be fairly weak, at least in the Swedish context, and this can also be deduced from the results in this article as the coefficients for relative household income were largely unchanged when introducing adolescent-level economic variables. More research is needed on the determinants of adolescents’ own economic and material conditions, for instance on the distribution of resources within families, their other income sources, consumption habits, and whether these differ based on characteristics such as gender, social class, or ethnicity.

This study is not without its limitations. First, the questions used to measure bullying victimization were related primarily to direct forms of bullying behavior. As the CILS4EU survey did not include questions regarding indirect- or cyber bullying victimization it remains for future research to address whether these, more indirect forms of bullying victimization, are associated with economic resources. A second limitation stems from the nature of cross-sectional data. While adverse peer relations are unlikely to affect household income, it is not as unlikely for peer rejection and bullying victimization to both be affected by economic and material deprivation and to affect adolescent perceptions of own experienced deprivation. More research on measures of own economic and material deprivation is needed to address this issue. Third, like all research using observational data, this study cannot entirely exclude the possibility that the observed patterns are generated by some other, here unobserved, characteristic or situation. Nevertheless, access to taxation and administrative register data made it possible to adjust for an extensive range of potential confounders using highly reliable measures and without introducing additional bias through selective non-response.

## Conclusion

The link between economic resources and adolescents’ peer relations has so far received limited research interest. This paper examines the association between economic resources and adverse peer relations using an adolescent-centered perspective of economic resources, involving both household income and own experienced economic and material assets, as well as two distinct forms of adverse peer relations: peer rejection and bullying victimization. The results showed that adolescents from households with a lower income than others in the same school were rejected by school class peers to a greater extent than students from more affluent households. Relative household income was not, however, found to be associated with bullying victimization; a finding largely in line with previous research using absolute measures of household economic resources (Olsson 2007). These results suggest that while low relative household income is associated with unfavorable relations with peers, this does not necessarily translate into the more severe outcome of being bullied. In contrast, adolescents’ own experienced economic deprivation—when related to problems of participation—was found to be associated with peer rejection and bullying victimization. Therefore, as far as peer rejection and bullying victimization is concerned, policies targeting the household economy are likely to be less efficient than policies directly targeting adolescents.

## Appendix

Table 3

**Table 3:** Regression of received rejection nominations (Models 1–3) and the probability of bullying victimization (Models 4–6) on relative household income, own economic and material deprivation, and control variables using casewise deletion instead of multiple imputation. Models 1–3 give OLS regression coefficients, whereas Models 4–6 give average marginal effects from logistic regressions

	Peer rejection (*n* = 3302)			Bullying victimization (*n* = 3929)		
	Model 1	Model 2	Model 3	Model 4	Model 5	Model 6
Household income (ref., Quintile 3)						
Quintile 1	0.747**	0.531**	0.529**	−0.003	−0.023	−0.023
	(0.159)	(0.179)	(0.179)	(0.019)	(0.020)	(0.020)
Quintile 2	0.356*	0.252	0.234	0.005	−0.007	−0.010
	(0.154)	(0.159)	(0.160)	(0.020)	(0.021)	(0.020)
Quintile 4	−0.041	0.019	0.022	0.008	0.013	0.015
	(0.137)	(0.137)	(0.135)	(0.019)	(0.020)	(0.020)
Quintile 5	−0.089	0.018	0.023	−0.012	−0.007	−0.004
	(0.152)	(0.152)	(0.154)	(0.017)	(0.017)	(0.017)
Not living with both parents		0.123	0.103		0.031*	0.028*
		(0.134)	(0.135)		(0.013)	(0.013)
Parental education (ref., Upper secondary)						
Comprehensive school or lower		0.355	0.312		0.009	0.006
		(0.288)	(0.288)		(0.031)	(0.029)
University		−0.256**	−0.239**		0.003	0.006
		(0.087)	(0.087)		(0.012)	(0.012)
Migration background (ref., Majority)						
Second generation		0.316*	0.268		−0.050**	−0.047**
		(0.135)	(0.150)		(0.013)	(0.013)
First generation, ≥ 2 years		0.197	0.083		0.007	0.001
		(0.202)	(0.228)		(0.028)	(0.025)
First generation, < 2 years		2.783**	2.770**		0.129	0.132
		(0.497)	(0.508)		(0.120)	(0.107)
Parent has disability pension		0.399*	0.355		0.049	0.038
		(0.195)	(0.196)		(0.028)	(0.025)
Parent is unemployed		0.360	0.343		0.015	0.014
		(0.200)	(0.203)		(0.025)	(0.025)
Misses activities due to lack of money			0.679*			0.097**
			(0.273)			(0.025)
Adolescent lacks cash margin			0.102			0.044**
			(0.101)			(0.011)
Adolescent lacks own room			0.314			0.002
			(0.223)			(0.022)
Own material resources (0–4)			0.023			−0.005
			(0.047)			(0.006)
Cognitive test score (centered)	−0.052**	−0.046**	−0.044**			
	(0.012)	(0.011)	(0.012)			
Girl	−0.340**	−0.344**	−0.339**	−0.006	−0.007	−0.011
	(0.121)	(0.121)	(0.121)	(0.012)	(0.012)	(0.012)
Age at interview (centered)	−0.228	−0.254	−0.262	0.007	0.004	0.004
	(0.137)	(0.139)	(0.137)	(0.016)	(0.016)	(0.015)
Constant	−0.239*	−0.239	−0.385*			
	(0.120)	(0.127)	(0.179)			
R-squared	0.036	0.053	0.060			

Robust standard errors in parentheses

**p* < 0.05, ***p* < 0.01

Table 4

**Table 4:** Bivariate Pearson correlation coefficients (weighted) among outcome and predictor variables in the analytical sample

	1	2	3	4	5	6	7
1 Bullying victimization	–						
2 Peer rejection	0.14**	–					
3 Relative household income	−0.01	−0.14**	–				
4 Misses out on activities	0.11**	0.10**	−0.05**	–			
5 Cash margin	0.09**	0.06**	−0.08**	0.15**	–		
6 Own room	0.00	0.07**	−0.11**	0.03	0.02	–	
7 Own material assets	−0.03	0.00	0.02	−0.07**	−0.17**	−0.14**	–

*N* = 3919; Correlations with peer rejection uses the rejection sub-sample (*N* = 3443)

**p* < .05, ***p* < .01

Table 5

**Table 5:** Regression of received rejection nominations (Model 3 and Model 3b) and probability of bullying victimization (Model 6 and Model 6b) on relative household income, own economic and material deprivation, and control variables. Model 3 and Model 6 are equivalent to the corresponding models in Table 2. Model 3b and Model 6b include additional control variables for internalizing and externalizing behaviour

	Peer rejection (*n* = 3895)		Bullying victimization (*n* = 4548)	
	Model 3	Model 3b	Model 6	Model 6b
Household income (ref., Quintile 3)				
Quintile 1	0.551**	0.564**	−0.011	−0.008
	(0.163)	(0.162)	(0.017)	(0.016)
Quintile 2	0.271	0.242	0.000	−0.006
	(0.157)	(0.155)	(0.018)	(0.017)
Quintile 4	0.010	0.024	0.016	0.019
	(0.133)	(0.133)	(0.018)	(0.017)
Quintile 5	−0.026	−0.016	0.002	0.004
	(0.153)	(0.151)	(0.016)	(0.015)
Not living with both parents	0.033	−0.050	0.028*	0.018
	(0.124)	(0.122)	(0.012)	(0.011)
Parental education (ref., Upper secondary)				
Comprehensive school or lower	0.191	0.195	−0.007	−0.014
	(0.249)	(0.248)	(0.024)	(0.023)
University	−0.205*	−0.198*	0.009	0.006
	(0.079)	(0.078)	(0.012)	(0.012)
Migration background (ref., Majority)				
Second generation	0.258	0.239	−0.042**	−0.038**
	(0.139)	(0.138)	(0.013)	(0.013)
First generation ≥ 2years	0.245	0.253	0.004	0.012
	(0.212)	(0.215)	(0.022)	(0.021)
First generation, < 2 years	2.304**	2.320**	0.122	0.080
	(0.463)	(0.465)	(0.123)	(0.093)
Parent has disability pension	0.457*	0.420*	0.031	0.026
	(0.194)	(0.194)	(0.024)	(0.023)
Parent is unemployed	0.328	0.285	0.001	−0.004
	(0.185)	(0.180)	(0.021)	(0.019)
Misses activities due to lack of money	0.673**	0.516*	0.109**	0.065**
	(0.253)	(0.254)	(0.027)	(0.023)
Adolescent lacks cash margin	0.092	0.103	0.040**	0.028**
	(0.097)	(0.097)	(0.011)	(0.011)
Adolescent lacks own room	0.267	0.278	0.007	0.008
	(0.216)	(0.219)	(0.024)	(0.023)
Own material resources (0–4)	0.017	−0.000	−0.006	−0.007
	(0.047)	(0.046)	(0.006)	(0.005)
Externalizing behaviour		0.645**		0.022
		(0.174)		(0.012)
Internalizing problems		0.155		0.095**
		(0.085)		(0.007)
Cognitive test score (centered)	−0.058**	−0.054**		
	(0.011)	(0.011)		
Girl	−0.306**	−0.348**	−0.013	−0.050**
	(0.117)	(0.120)	(0.012)	(0.012)
Age at interview (centered)	−0.230	−0.232	0.002	0.003
	(0.124)	(0.124)	(0.014)	(0.014)
Constant	−0.322	−0.496**		
	(0.178)	(0.181)		

Robust standard errors in parentheses

**p* < 0.05, ***p* < 0.01

## Footnotes

1 For a small number of observations, responses from Wave 2 and Wave 3 were used to replace missing data on stable characteristics (e.g., date and country of birth, gender).
